# Alexithymia and Autism Spectrum Disorder: A Complex Relationship

**DOI:** 10.3389/fpsyg.2018.01196

**Published:** 2018-07-17

**Authors:** Jessie Poquérusse, Luigi Pastore, Sara Dellantonio, Gianluca Esposito

**Affiliations:** ^1^Department of Psychology and Cognitive Sciences, University of Trento, Rovereto, Italy; ^2^Montreal Neurological Institute, McGill University, Montreal, QC, Canada; ^3^Department of Education, Psychology, Communication, University of Bari, Bari, Italy; ^4^Psychology Program, School of Social Sciences, Nanyang Technological University, Singapore, Singapore

**Keywords:** alexithymia, autism spectrum disorders, autism, ASD etiology, personality

## Abstract

Alexithymia is a personality construct characterized by altered emotional awareness which has been gaining diagnostic prevalence in a range of neuropsychiatric disorders, with notably high rates of overlap with autism spectrum disorder (ASD). However, the nature of its role in ASD symptomatology remains elusive. Here, we distill research at the intersection of alexithymia and ASD. After a brief synopsis of the studies that played a pioneering role in the identification of the overlapping fields between alexithymia and ASD, we comb the literature for evidence of its overlap with ASD in terms of prevalence, etiology, and behaviors. Through a formalized framework of the process of emotional interpretation and expression, we explore evidence for where and how deficits arise in this complex network of events. We portray how these relate to the dynamic interplay between alexithymic and autistic traits and find emerging evidence that alexithymia is both a cause and consequence of autistic behaviors. We end with a strategic proposal for future research and interventions to dampen the impacts of alexithymia in ASD.

## Introduction

Over the last two decades the relationship between alexithymia and autism spectrum disorder (ASD) has gained significant attention – there has been a surge in the number of studies aimed at investigating the relationship between these conditions, including from a conceptual and etiological point of view, as well as with regards to the implications of this relationship for clinical and therapeutic practices. Alexithymia is highly prevalent and plays an important and complex role in ASD, with approximately half of individuals with ASD estimated as having alexithymia, but the nature of its role remains elusive. Empirical evidence of the biological basis of alexithymia in ASD is suggestive of its role as a root cause, while empirical evidence of alexithymia as a byproduct of core ASD deficits is suggestive, conversely, of its role as consequence of ASD. It may also play a role as both cause and consequence of ASD in a feedforward cycle between alexithymia and ASD symptomatology.

Here, we review existing literature on alexithymia and its relationship to ASD. Our first aim is to provide a brief synopsis of the research on the relationship between ASD and alexithymia, including clarifying when and how they originate. Next, we will illustrate the prevalence of alexithymia in ASD as well as their overlap in terms of etiology and features, propose both empirically and theoretically driven causal and consequential roles for alexithymia in ASD, and suggest clinically useful constructs and interventions as well as specific areas for beneficial future investigation.

## Autism Spectrum Disorder

Autism spectrum disorder has long been well-known and has a stable definition in psychiatric nosography. Initially, the typical characteristics of this disease were identified by Eugen Bleuler as early symptoms of schizophrenia, or by Melanie Klein of psychosis (Wing, 1997; Wolff, 2004). The current meaning of the term ‘autism’ was developed by and can be attributed to Asperger (1938, 1944), while the lemma ‘infantile autism’ was introduced by Kanner (1943). Asperger and Kanner used the term to indicate a disorder of organic origin with severe behavioral, affective, communication and social skills impairment, characterized by little interest in others, speech disorder, attention deficit and compulsive and repetitive behavior. Since the early 1980s – and especially after the publication of the DSM-III (American Psychiatric Association [APA], 1980) – it has been recognized as an autonomous pathological condition definitively differentiated from schizophrenia. Eventually, descriptions of the condition were slightly modified and accurate criteria for its assessment were provided in 1987 by the DSM-III-R (American Psychiatric Association [APA], 1987). The publication of the DSM-IV (American Psychiatric Association [APA], 1994) placed autism in the wider category of pervasive developmental disorders, a complex of syndromes that affect social interaction, communication and the capacity to develop varied interests. In this new conceptualization, a milder form of autism, Asperger’s syndrome, was distinguished, in which mental retardation and linguistic impairment are less severe. With the publication of the DSM-IV and later of the DSM-V (American Psychiatric Association [APA], 2013), a view of autism was gradually developed based on the idea of a spectrum: autism is no longer seen as a categorical condition, but is understood instead in terms of continuous traits, hence the adoption of the term ASD. In this view, the condition is characterized by the co-occurrence of various psychological disorders due to underlying neuropsychological and functional impairments. Historically, many hypotheses have been put forth on the origin of autism. Though some of them – mainly in the realm of psychoanalytic research – have suggested both a relational and environmental origin, it is now clear that genetic factors play a predominant role in its etiology (Colvert et al., 2015).

## Alexithymia

Unlike ASD, alexithymia does not have a steady classification in the psychiatric nosography. Alexithymia was first introduced into the lexicon of psychiatry by Peter E. Sifneos in the early 1970s (Sifneos, 1973) to characterize a number of patients with psychosomatic complaints who were being treated by various psychoanalysis research groups across Europe and North America (Sifneos, 2000). Literally, it indicates the lack of terms to express emotions and moods (a: lack; lexis: word; thymos: mood or emotion, see Lesser, 1981). In fact, a common trait in these patients was their inability to verbalize their emotions, either due to their unawareness of the feelings that corresponded to these emotions or due to their confusion of emotional and bodily feelings. Indeed, they would typically describe their emotional experience in terms of the somatic sensations they incurred, reflecting the so called “operatory thinking” which had already been described by Marty and de M’Uzan (1963) and Marty et al. (1963). Their incapacity to speak of their emotions was further accompanied by an impoverished narrative style, especially in the use of figures of speech and metaphors, and by a characteristic aprosodia, as if the emotional experience were uninteresting and extraneous to them.

Sifneos originally hypothesized that the patients exhibiting these symptoms suffered from a particular form of linguistic impairment due to an “emotional agnosia” (Sifneos, 1967); later he suggested that these symptoms should be rather interpreted in terms of a “feeling aphasia” (Sifneos, 1996). While Sifneos was originally open to the possibility of considering alexithymia as a pathological personality trait causally related to psychosomatic illness (Sifneos, 1974), later research challenged the idea of alexithymia as a single diagnostic category since characteristic aspects of alexithymia were seen among non-psychosomatic clinical populations as well. Further research actually showed that alexithymic traits could be observed in people suffering from very different clinical conditions such as neurodegenerative diseases (Sturm and Levenson, 2011; Ricciardi et al., 2015), psychiatric conditions such as depression and suicidality (Honkalakampi et al., 2000; Hintikka et al., 2004) – though the interplay between affective and cognitive impairments in the etiology of depression is still a point of interesting contention (Gonda et al., 2015), schizophrenia (Fogeley et al., 2014), eating disorders (Nowakowski et al., 2013), and ASD (Bird and Cook, 2013). Moreover, 10% of the non-clinical population exhibits some alexithymic traits (Salminen et al., 1999).

Today alexithymia is considered a “sub-clinical phenomenon” (Silani et al., 2008), not identifying a personality disorder *per se*, but a personality trait with a dimensional nature (Taylor et al., 1991; Taylor, 1994). This is characterized by an impairment in the awareness of emotions due to a deficit in processing of affective information (Vermeulen et al., 2006). The main characteristics of alexithymia include (1) difficulty identifying feelings and distinguishing between feelings and bodily sensations of emotional arousal, (2) difficulty describing feelings to other people, (3) reduced capacity to fantasize and to imagine, (4) stimulus-bound, externally oriented cognitive style (Nemiah et al., 1976; Krystal, 1988; Taylor et al., 1997; Timoney and Holder, 2013), and, more recently, (5) low perspective-taking as well as difficulty understanding and describing the emotions of others (Saymur et al., 2013).

The lack of emotional awareness has a negative impact on subjective emotion regulation (Connelly and Deney, 2007) and compromises the understanding of others’ emotions, giving rise to problems in social interaction. In particular, because of their difficulty identifying and classifying feelings, people suffering from alexithymia cannot interpret or recognize emotional stimuli (e.g., facial expression or tone of voice), both verbal and nonverbal (Spitzer et al., 2005; Vanheule et al., 2007; Megank et al., 2009). As a consequence, they have difficulty establishing social relationships characterized by intimacy and proximity, understanding the intentions and attitudes of others, and making morally relevant decisions that take into account others’ points of view. These aspects of alexithymia together with the communication and social skills deficits are among the most relevant overlapping elements between alexithymia and ASD.

The idea of a relationship between alexithymia and ASD was recently developed, starting from the observation that both types of patients exhibit similar social difficulties. This phenomenon established itself in the context of psychological research as a result of the development of the construct of emotional intelligence by Salovey and Mayer (1990) and Goleman (1996), which focuses on the links between emotion and cognition, critical to the development of emotional competence (Saarni, 1999). Emotional intelligence and emotional competence are similar constructs that are used to investigate the capacity to detect, comprehend and logically organize emotional information concerning oneself and others, together with the capacity to manage and regulate behavior in social interactions. It is in this line of study that alexithymia and ASD began to be considered as conditions characterized by widely overlapping traits related to social difficulties and deficits in emotional competence.

## The Intersection Between Alexithymia and ASD

The idea that alexithymia and ASD are somehow related was first considered in the mid-1990s. At the time, several clinical studies were performed on groups of people suffering from eating disorders (especially anorexia nervosa). On one hand, they highlighted the co-occurrence of these disorders with impairments in social competence, while on the other hand, they stressed the co-occurrence of an impairment of social competence with some traits that were considered typical of ASD, such as empathy disorders (cf. Gillberg, 1992), as well as others that were considered typical of alexithymia, such as the difficulty expressing emotions verbally, identifying feelings and distinguishing emotions from somatic sensations (cf. i.a. Gillberg et al., 1995; Rastam et al., 1997; De la Rubia and Rojas, 2001; Soya and Tenaglia, 2001). In a short time, the relationship between alexithymia and ASD started to be investigated more widely, including with respect to aspects not related to compromised social competence. Other areas of intersection were identified, such as disorders of cognitive functioning, impaired self-awareness and mentalization, poor linguistic mastery and difficulty with behavior control (Corcos, 2003; Fitzgerald and Bellgrove, 2006; Hill and Berthoz, 2006). This has led to greater attention on the incidence of alexithymic traits in clinical populations suffering from ASD and on the assessment that about half of people suffering from ASD exhibit some relevant alexithymic traits (Hill et al., 2004; Berthoz and Hill, 2005; Bird and Cook, 2013).

The cognitive, linguistic and behavioral issues of people with ASD have long been known (cf. i.a. Hermelin and O’Connor, 1970; Baron-Cohen et al., 1985; Denckla, 1986; Frith, 1991; Rogers and Pennington, 1991; Tager-Flusberg, 1992; Yirmiya et al., 1992; Rapin and Dunn, 1997). However, in this context, research has focused more specifically on the co-occurrence of these issues with specific characteristics typical of alexithymia such as the difficulty identifying and attributing emotions (Hill et al., 2004; Moriguchi et al., 2006; Silani et al., 2008; Cook et al., 2013), an excessively pragmatic and utilitarian thinking style (Patil et al., 2016), poor emotional lexicon (Lartseva et al., 2015), difficulties understanding metaphorical language (Wotschak and Klann-Delius, 2013), issues with the interpretation of nonverbal clues (Bird et al., 2011), aprosody (Heaton et al., 2012), difficulties with emotion regulation and emotional expression (Weiss et al., 2014; Costa et al., 2017), and difficulty discriminating bodily sensations due to an altered somatic sensibility (Liss et al., 2008; Shah et al., 2016; Gaigg et al., 2018).

In spite of the high volume of research devoted to the relationship between alexithymia and ASD, no one unequivocal answer has been provided to the question surrounding their precise relationship. In fact, it is still not clear whether this should be interpreted in causal terms (and in which direction), whether one of them is a secondary phenomenon due to the occurrence of dysfunctions caused by the onset of the other (i.e., whether there is a comorbidity or an epiphenomenal relationship), or whether it is a mere, yet unspecified, co-occurrence. This situation of etiological uncertainty is partly due to our incomplete understanding of the cognitive, physiological, and neurophysiological mechanisms underlying the onset of both conditions. It is thus of particular importance to assess our current knowledge of their relationship and to continue to analyze the literature for new clues in this direction.

## Evidence of Diagnostic, Etiological, and Phenotypic Overlap Between Alexithymia and ASD

Alexithymia is common in ASD, both low and high-functioning (Hill et al., 2004; Fitzgerald and Bellgrove, 2006; Paula-Pérez et al., 2010; Griffin et al., 2016). It is also more prevalent in relatives of individuals with ASD, potentially constituting an element of the broader autism phenotype found in such relatives (Szatmari et al., 2008). Etiologically, research on ASD and alexithymia suggest both broad genetic and neurobiological overlap, including oxytonergic and serotonergic system activation and amygdala, cingulate, and prefrontal cortex functioning (Elagoz Yuksel et al., 2016; Muller et al., 2016; Donovan and Basson, 2017). Most importantly however, there is also much trait overlap between alexithymia and ASD. Individuals with alexithymia have difficulty in both the verbal and the nonverbal identification of emotions: they are unable to describe their feelings, use emotion terms, or recognize emotions in facial expressions and other nonverbal emotional stimuli such as tones of voice or situations with strong emotional connotations. They often confuse emotions with somatic sensations. In parallel, individuals with ASD have difficulty with the cognitive processing of their emotions, identifying, and describing feelings (Hill et al., 2004; Shah et al., 2016). More specifically, they have difficulty with general emotional competence, including emotion perception, recognition and regulation, particularly facial emotion recognition, but also recognition of emotional tones of speech and prosody, verbal content, and body movement, including the coordination or integration thereof (Serafini et al., 2017; Gaigg et al., 2018). It is important to note here that these deficits may, however, be linked to the deficits generally seen in speech and language competence in individuals with ASD (South and Rodgers, 2017) and as they relate to emotions in alexithymia (Allen et al., 2013). Similar and consequent, but not identical to the above, another area of overlap is social, in individuals’ relationship and response to others, including cognitive and emotional states, notably in the form of cognitive and affective empathy. Individuals with ASD and alexithymia display reduced levels of enjoyment of prosocial interactions (Gebauer et al., 2014). Evidence is also substantial that individuals with ASD and alexithymia display decreased levels of empathy (Luminet et al., 2006; Lartseva et al., 2015), possibly linked to neurobiological deficits in limbic and paralimbic neural activity responses to emotionally salient stimuli (Moriguchi and Komaki, 2013). Connected to empathy, a concrete, utilitarian thinking style, not dissimilar to “operative thinking,” is common in alexithymia (Suslow and Junghanns, 2002) and ASD, though the roles of this type of thinking on moral judgment has been suggested to be different in alexithymia as compared to ASD (Lemche et al., 2004). Clearly, alexithymia and ASD share many overlapping features in emotional, social, cognitive – verbal and nonverbal – realms, with varying degrees of convergent consequences on their individual and social behavior and lives. This lays the foundation for investigating the nature of their complex relationship in the context of emotional processing.

## Mechanistic Links in Emotional Processing Between Alexithymia and ASD

To better hone in on where the deficits arise in alexithymia and ASD, we break down the phenomenon of emotional processing and, at each level, we review evidence of a deficit and how this may shed light on the role of alexithymia in ASD. Briefly, the steps we define are (1) interpreting one’s emotions – through interoception, affective and cognitive manifestations, (2) responding to and regulating one’s emotions – verbally and nonverbally, and (3) appropriately interpreting and responding to others’ emotions.

### Interpreting Emotion in the Self – General Emotional Awareness, Recognition of Sensory Manifestations of Emotion, and Cognitive Appraisal Thereof

A factor that plays a relevant role in our emotion perception and thus in emotion understanding is interoceptive awareness: through it, we become capable of identifying, distinguishing, and assessing the physiological activations related to our emotions. Several studies show that the mechanism responsible for our emotion awareness have significant overlaps with the neural systems that support our interoceptive awareness (Damasio et al., 2000; Critchley et al., 2004; Pollatos et al., 2007). The general notion of interoceptive awareness has been further specified by distinguishing two underlying different, and partially independent, capacities that are included in it: the so-called interoceptive accuracy and interoceptive sensibility (Garfinkel and Critchley, 2013; Garfinkel et al., 2015). The notion of interoceptive accuracy describes our capacity to identify internal body sensations, while the notion of interoceptive sensibility refers to our capacity to focus on our internal sensations and to take them into consideration, including from a cognitive point of view.

This distinction is relevant in order to understand the relationship between alexithymia and ASD since the difficulty in recognizing and distinguishing physiological activations is a trait typical of alexithymia while atypical forms of sensory perception are characteristic of people suffering from ASD. Some studies show that alexithymics exhibit an excessive activation with respect to the physiological component of emotional arousal (Kano and Fukudo, 2013) and an atypical interoceptive awareness (Herbert et al., 2011; Ernst et al., 2014) which can be interpreted as the result of an increased interoceptive accuracy which is not accompanied by an adequate interoceptive sensibility. Atypical forms of interoceptive awareness were observed in people suffering from both alexithymia and ASD (Brewer et al., 2016): since interoceptive accuracy is mainly related to difficulties discriminating among interoceptive signals typical of alexithymia, it was suggested that alexithymia might be responsible for the atypical interoception manifested in people suffering from ASD, and that this might reflect itself in their difficulties understanding their bodily states as well as their and others’ emotions (Lombardo et al., 2007; Cook et al., 2013). Interestingly, this interoceptive deficit may be linked to weak theory of mind, a common feature of ASD, since an inability to understand one’s own complex inner states may extend to and manifest itself as an inability to understand those of others. This said, results are controversial and some studies found that difficulties in emotional awareness were related neither to impairments in self-reflection nor mentalizing (Silani et al., 2008; Bird and Viding, 2014), while others have shown only partial correlation between theory of mind, emotion perception and the cognitive understanding of the emotion of others, i.e., perspective taking (Moriguchi et al., 2006; Oakley et al., 2016).

Altered interoceptive sensitivity observed in alexithymia could have implications at a cognitive level (interoceptive sensibility), as well as at a metacognitive level (general interoceptive awareness), inducing difficulties in making sense of one’s sensory experiences, either due to a core sensory processing deficit or to sensory overload as a consequence of inadequate filtering. The presence of alexithymia in people with ASD may in fact involve a disruption in how physiological arousal modulates the subjective experience of feelings (Gaigg et al., 2018). Other studies have outlined how alexithymia in people with ASD may be interpreted as a consequence of the extreme sensory processing patterns of ASD (Serafini et al., 2017), and, more specifically, of the atypical sensory function and associated intolerance of uncertainty, possibly due to a deficit in limbic-, insula-, and medial prefrontal cortex-based network integration (South and Rodgers, 2017). This said, since significant data points to deficits in the synthetic interpretation of physiological responses to emotion, and implied processing of large-scale information flow, it may be of particular interest to explore the link with ASD theories of weak central coherence and executive dysfunction.

### Responding to and Regulating Emotion in the Self – Expression and Action, Verbal, and Nonverbal

We have seen that deficits in alexithymia can arise at the level of emotional understanding, but many difficulties may be linked to emotional expression or externalization. Whether an emotion has been consciously recognized or not, alexithymia and its linked disturbances in ASD may reflect a mismatch between the affective and expressive – including linguistic, visual artistic, musical – systems.

An example of emotional reactivity in alexithymia, emotional responsiveness to music and speech prosody provides a powerful lens – arguably representative of responses to other emotionally salient stimuli – into the emotional processing disruptions seen in alexithymia and ASD. Alexithymia, not lack of emotional responsiveness to music, may be the cause of reduced verbal responsiveness to music in ASD – indeed, though results are controversial in alexithymia, and some studies measuring autonomic reactions to emotionally salient stimuli showed evidence of both hyper-arousal, as per blunted sympathetic activation in response to anger recall (Neumann et al., 2004), and hypo-arousal, as per heightened blood pressure responses to anger provocation (Waldstein et al., 2002), overall physiological responsiveness to the emotionally salient stimuli of music among ASD individuals is intact (Allen et al., 2013), as is recognition of emotion in music (Quintin et al., 2011; Gebauer et al., 2014), suggesting the difficulty comes from the disjunction, and associated complications, between emotional understanding and expression. This is consistent with solid evidence and discussion over the last few decades on the hypothesis that alexithymia is generated by a decoupling between physiological, cognitive, and sometime expressive responses to emotionally salient stimuli, i.e., broadly between physiological arousal and subjective experience (Stone and Nielson, 2001; Eastabrook et al., 2013). Indeed, consistent with the above, the emotional impairments in ASD gravitate around the emotional language processing system (Lartseva et al., 2015), again highlighting alexithymia, or the lack of words to express emotions, as a causative factor of ASD. More specifically, while individuals with ASD are able to correctly classify emotional language stimuli as emotionally positive or negative, they are unable to explain their choices in further depth and display atypical patterns of attention and memory performance, as well as abnormal physiological and neural activity – they show deficits in recalling emotional material and in semantically processing emotional content (Luminet et al., 2006) and are less primed by emotional contexts to process emotional words more easily (Suslow and Junghanns, 2002). More broadly, they have deficits in general emotional and internal state vocabulary (Lemche et al., 2004) and the fact that they have difficulty perceiving and processing speech prosody or melody of speech that has emotional content (Goerlich-Dobre et al., 2014) confirms their difficulty with general emotional language, as it relates to themselves and others. This may be due to the increased cognitive load required to process emotionality in music, as evidence by increased neural activity in emotional music-reactive brain regions (Gebauer et al., 2014). While differences remain inconsistent (Swart et al., 2009; Bhatara et al., 2010), it seems clear that broad brushstrokes of emotional verbalization are conserved while subtleties are not, and the core deficit in alexithymia seems to be at the level of detailed symbolization – largely through verbal conceptualization – of emotional experience. This supports its role as causative factor of ASD symptomatology.

Though less common, emotions may naturally also be expressed in the form of nonverbal expression, of particular relevance in the context of ASD, which is replete with differences in sensory and cognitive perception and expression, of the self and the external world (Markram and Markram, 2010). Differences in nonverbal emotional expression at the level of body posture and facial expression are seen in ASD, with evidence of abnormalities in emotional facial expression which may be explained by co-occurring alexithymia (Trevisan et al., 2016). Art allows individuals with alexithymia to express themselves and potentially access the verbal expression system which they need to process and vent their emotions (Meijer-Degen and Lansen, 2006); interestingly, individuals with ASD have high rates of synesthesia and often use colors to express their emotions (Neckar and Bob, 2017). Dance, or rhythmic movement therapy, is another effective outlet for symptoms of alexithymia (Malkina-Pykh, 2013) and may be intricately linked to emotional competence (Bojner Horwitz et al., 2015). As mentioned above, music is another alternate vehicle for understanding, processing, and communicating emotions (Allen and Heaton, 2010; Zangwill, 2013). Individuals with ASD have intact or superior musical pitch processing and are able to properly identify the positive or negative emotional valence of music stimuli. As seen above, their responsiveness to emotionality in music is complex, however, and reflected in a network of both intact and altered brain circuit responses to emotional processing of music (Caria et al., 2011). This evidence of nonverbal emotional expression as an outlet for individuals with alexithymia and ASD further suggests that alexithymia may be a causative factor for some of the emotional symptoms of ASD, but can be compensated for through alternate means of emotional interoception and expression. Indeed, an important part of responding to emotion is at the level of action planning, or strategizing a response plan (usually subconsciously) to difficult emotions (Samson et al., 2015b), and when provided with such a strategy, individuals with ASD respond well, highlighting the tremendous potential, through training, to help individuals with ASD understand and deal with their emotions in a healthy way.

The above responses to one’s emotions represent a crucial step to emotional regulation – expression of emotional conflicts may be a first avenue toward their subsequent recognition and regulation. In addition, an important component of emotional processing is being able to regulate emotions within and to adapt to various contexts. This is particularly important in the context of ASD, since a frequent feature of ASD is behaviors reflecting a lack of sound emotional regulation, with many individuals with ASD displaying uncontrollable tantrums and outburst and self-harm behaviors (Samson et al., 2014). Interestingly, behavioral suppression strategies that focus on inhibiting emotion-expressing behavior has been shown to be the strategy used by individuals with ASD, who naturally favor emotional expressive suppression over cognitive reappraisal (Samson et al., 2012, 2015a,b). This is highly similar to alexithymia, in which individuals rely more on suppressing than reappraising their emotions (Swart et al., 2009). Though contentious, as per scarce previous studies which may have failed to analyze the effect of alexithymia as a whole rather than through its subdimensions (Samson et al., 2012), this may point to further support for the alexithymia hypothesis whereby alexithymic features may be at the core of the similarly manifested emotional regulation difficulties seen in ASD. While still important, regardless of the nature of the interaction between alexithymia and ASD in the context of emotional regulation, it is important to note, and incorporate into treatment plans, that strategies that focus on situational reappraisal, by creative a narrative that down-regulates the impact of negative emotions, are a significantly more adaptive long-term strategy (Gross, 2015).

### Interpreting and Responding to Emotion in Others – Expression and Action, Immediate, and Subsequent

A final important step, or aspect, of emotional processing, is in understanding of the self and others, and appropriate response to the valuable information about the human and non-human external world that emotions provide. These challenges generalize beyond the self to the emotions of others, with repercussions on how individuals behave socially. Not surprisingly, individuals with alexithymia have difficulties interpreting and describing the emotional and cognitive states of others. These difficulties manifest themselves as deficits in emotional mentalizing (Moriguchi et al., 2006; Swart et al., 2009), poorer recognition of emotional expression in faces (Grynberg et al., 2012; Cook et al., 2013), deficits in the recognition of vocal affect (Heaton et al., 2012), and alexithymia-induced deficits in eye fixation, a rich source of information regarding emotional state (Bird et al., 2011; Lee and Anderson, 2017). In addition, levels of alexithymia correlate negatively with models-of-self but also models-of-others, and alexithymics show decreased emotional contagion (i.e., mentalization of affects reflected in autonomic muscular activity) in response to others’ facial responses (i.e., somatic affects), as seen as deficits in facial muscle emotional processing as measured by somato-motor electromyographic activity (Sonnby-Borgström, 2009; Scarpazza et al., 2018). Consistently, stronger alexithymia and lower capacity for self-memory are predictive of larger mentalizing impairments and autism quotient scores (Lombardo et al., 2007), which may provide an interesting link to the cognitive deficits in theory of mind and cognitive and affective aspects of differences in empathy seen in ASD.

Interestingly, moral decision-making, which generally incorporates rational and emotional insights, is less subject to emotional biases in individuals with ASD due to the presence of alexithymia (Brewer et al., 2015). This was corroborated by a study which found no differences in moral decision-making in individuals with both ASD and alexithymia – while ASD reduced utilitarian bias due to elevated distress in social situation, alexithymia increased utilitarian bias due to reduced empathic concern (Patil et al., 2016). This may be consistent with the fact that alexithymia decreases altruism in real social decisions (Feldmanhall et al., 2013). Finally, other consequences of alexithymia on social interaction include compromised social assertiveness (Roelofs et al., 2015) and reduced interest in people and shared interests – consistently, empathic brain activity in the anterior insula was predicted by alexithymia, not ASD (Bird et al., 2010), and the degree of anterior insula activity has been correlated to individuals’ self-reported degree of alexithymia and empathy (Silani et al., 2008).

As such, alexithymia may break down a rung in the ladder of internal and external emotional reaction in individuals with ASD, impinging on their ability to appropriately respond to emotion. Our balanced synopsis seems to support the ‘alexithymia hypothesis’ (Bird and Cook, 2013) that posits that alexithymia, not ASD, is a leading cause of the emotional deficits inherent to ASD symptomatology.

## Synthetic Perspective

To best inform therapeutic strategies, it will be critical to better structure our understanding of the etiologies of and relationship between alexithymia and ASD. Emerging overlapping etiologies between alexithymia and ASD will be of particular interest, including the potential association of certain subtypes of alexithymia with ASD. In addition, it will be important to keep in mind the peripheral, and more indirect, links between alexithymia and ASD. Alexithymia in itself may cause anxiety and related sleep issues (Tani et al., 2004), and the inability to healthily express and externalize emotions could lead to a variety of psychosomatic manifestations which may be manifested as immune, gastrointestinal, and circadian disruptions, all frequently seen in ASD.

Undoubtedly, alexithymia is a heuristically useful concept for clinicians and non-clinicians alike. Alexithymia is a negative prognostic factor for health and psychotherapy outcomes (Kojima, 2012), and emotion processing difficulties have been linked to depression in ASD (Hill et al., 2004), making it important to recognize and treat alexithymic traits as soon and as best as possible. Central to the concept of neurodiversity, it will be important to let this recognition of alexithymia guide clinical and non-clinical care – since there is potential for training individuals with alexithymia to better recognize emotional expressions in faces (Cook et al., 2013) and have better recollection of emotional memories (Luminet et al., 2006), there is tremendous potential for providing the tools for effectively coping with, strategizing with, and coherently expressing emotions that may not be obvious to individuals with alexithymia or ASD (Costa et al., 2017), including compensatory intellectual strategies, nonverbal reasoning skills and re-learning the rules of socio-moral norms (Patil et al., 2016). Cognitive reappraisal of emotionally salient situations, a response which is not natural, but easily learned in individuals with alexithymia and ASD, is a further example of a concrete, actionable intervention with positive health outcomes (Gross, 2015). Further examples of effective therapy will thus include cognitive behavioral therapy (Spek et al., 2008), but also mindfulness-based therapies that encourage heightened interoceptive sensitivity and accuracy (Gaigg et al., 2018) as well as more non-traditional forms of therapy tailored to individual modes of perception and communication, including verbal, artistic, musical, and kinesthetic expression. Since evidence is only growing of the benefits of alternate, nonverbal, forms of expression in alleviating alexithymia-linked challenges (Heiman et al., 1994), it is most important to first recognize alexithymia to be able to then invest in developing and implementing creative therapies and original systems of communication that are tailored to an individual’s mode of perception and experience. Adopting such changes will hopefully provide an extra step toward the full adoption and embracement of inclusive neurodiversity, during which our thinking about alexithymia, including as it relates to ASD, must keep being increasingly creative and multidimensional as we develop unique individualized strategies.

## Author Contributions

JP, LP, SD, and GE: conceived, designed and wrote the paper.

## Conflict of Interest Statement

The authors declare that the research was conducted in the absence of any commercial or financial relationships that could be construed as a potential conflict of interest.
