# Corrigendum: Alexithymia and Autism Spectrum Disorder: A Complex Relationship

**DOI:** 10.3389/fpsyg.2018.01638

**Published:** 2018-09-05

**Authors:** Jessie Poquérusse, Luigi Pastore, Sara Dellantonio, Gianluca Esposito

**Affiliations:** ^1^Department of Psychology and Cognitive Sciences, University of Trento, Rovereto, Italy; ^2^Montreal Neurological Institute, McGill University, Montreal, QC, Canada; ^3^Department of Education, Psychology, Communication, University of Bari, Bari, Italy; ^4^Psychology Program, School of Social Sciences, Nanyang Technological University, Singapore, Singapore

**Keywords:** alexithymia, autism spectrum disorders, autism, ASD etiology, personality

In the original article, there was an error. A typo was found in the Abstract - the word “played” was misspelled.

A correction has been made to the ***Abstract***:

Alexithymia is a personality construct characterized by altered emotional awareness which has been gaining diagnostic prevalence in a range of neuropsychiatric disorders, with notably high rates of overlap with autism spectrum disorder (ASD). However, the nature of its role in ASD symptomatology remains elusive. Here, we distill research at the intersection of alexithymia and ASD. After a brief synopsis of the studies that played a pioneering role in the identification of the overlapping fields between alexithymia and ASD, we comb the literature for evidence of its overlap with ASD in terms of prevalence, etiology, and behaviors. Through a formalized framework of the process of emotional interpretation and expression, we explore evidence for where and how deficits arise in this complex network of events. We portray how these relate to the dynamic interplay between alexithymic and autistic traits and find emerging evidence that alexithymia is both a cause and consequence of autistic behaviors. We end with a strategic proposal for future research and interventions to dampen the impacts of alexithymia in ASD.

The authors apologize for this error and state that this does not change the scientific conclusions of the article in any way.

The original article has been updated.

## Conflict of interest statement

The authors declare that the research was conducted in the absence of any commercial or financial relationships that could be construed as a potential conflict of interest.

